# Detection of *Babesia* spp. in High Altitude Cattle in Ecuador, Possible Evidence of the Adaptation of Vectors and Diseases to New Climatic Conditions

**DOI:** 10.3390/pathogens10121593

**Published:** 2021-12-08

**Authors:** María A. Chávez-Larrea, Cristina Cholota-Iza, Viviana Medina-Naranjo, Michelle Yugcha-Díaz, Jorge Ron-Román, Sarah Martin-Solano, Gelacio Gómez-Mendoza, Claude Saegerman, Armando Reyna-Bello

**Affiliations:** 1 Research Unit of Epidemiology and Risk Analysis Applied to Veterinary Sciences (UREAR-ULiège), Fundamental and Applied Research for Animal and Health (FARAH) Center, Department of Infections and Parasitic Diseases, Faculty of Veterinary Medicine, University of Liège, 4000 Liège, Belgium; machavezlarrea@student.uliege.be; 2 Grupo de Investigación en Sanidad Animal y Humana (GISAH), Carrera de Ingeniería en Biotecnología, Departamento de Ciencias de la Vida y la Agricultura, Universidad de las Fuerzas Armadas-ESPE, Sangolquí P.O. Box 171-5-231, Ecuador; cecholota@espe.edu.ec (C.C.-I.); vivi.1292@live.com (V.M.-N.); emyugcha@espe.edu.ec (M.Y.-D.); ssmartin@espe.edu.ec (S.M.-S.); 3 Grupo de Investigación en Sanidad Animal y Humana (GISAH), Carrera de Ingeniería Agropecuaria, Departamento de Ciencias de la Vida y la Agricultura, Universidad de las Fuerzas Armadas-ESPE, Sangolquí P.O. Box 171-5-231, Ecuador; jwron@espe.edu.ec; 4 Carrera de Ingeniería Agropecuaria, Departamento de Ciencias de la Vida y la Agricultura Extensión Santo Domingo, Universidad de las Fuerzas Armadas-ESPE, Sangolquí P.O. Box 171-5-231, Ecuador; gagomez@espe.edu.ec

**Keywords:** babesiosis, *Babesia bovis*, *Babesia bigemina*, cattle, *R. microplus*

## Abstract

Background: Babesia species are intraerythrocytic protozoa, distributed in tropical and subtropical areas of the world, causing anemic diseases in many animals, including cattle. This disease, called babesisosis, is transmitted from one animal to another through ticks (Tick Borne-Disease or TBD). On the other hand, Ecuador has a tropical climate that allows the development of the vector *Rhipicephalus microplus*, and therefore favors the transmission of *Babesia* spp. in cattle. Methods and principal findings: We determined the presence of *Babesia* spp. by PCR using 18s ribosomal gene as target (18s PCR) in 20 farms in the area of El Carmen (zone below 300 m above sea level) and 1 farm in Quito (2469 m.a.s.l.). In addition, we analyzed parameters such as age, sex, and packed cell volume (PCV) as explanatory variable associated with the disease. Results: The 18s PCR test showed that 18.94% (14.77% *Babesia bovis* and 4.17% *Babesia bigemina*) and 20.28% (14.69% *B. bovis* and 5.59% *B. bigemina*) of the cattle were positive for *Babesia* spp in farms sampled in El Carmen and in Quito, respectively. Age influenced the presence of animals positive for *Babesia* spp., but sex and PCV did not. The phylogenetic analysis of sequences showed 4 isolates of *B. bovis* and 3 isolates of *B. bigemina* in the 2 study zones, with similarities between 99.73 and 100% with other sequences. One *B. bovis* isolate was similar in the zone of El Carmen and Quito. Conclusion and significance: This work is the first molecular characterization of *B. bigemina* and *B. bovis* in Ecuador, and it is also the first evidence of *Babesia* spp. in cattle in the area of Quito at an altitude of 2469 m.a.s.l., being the highest altitude reported for animals with babesiosis and for the tick *R. microplus*. Climatic factors as well as mobility of tick-carrying animals without any control allow the presence of Babesiosis outbreaks in new geographical areas.

## 1. Introduction

Babesiosis, a disease caused by intraerythrocytic protozoa of the genus *Babesia* spp., affects a wide variety of domestic and wild animals and even birds [1]. Two species, *Babesia bovis* and *Babesia bigemina*, are present in cattle in Central and South America [2]. The main clinical manifestations in cattle are fever, anemia, hemoglobinuria, and nervous signs associated with sequestration of infected erythrocytes in the microcapillaries of the brain [2,3].

Bovine babesiosis is found in Latin American countries such as Venezuela, Colombia, Peru, and Brazil [4,5,6,7]. In the state of Santa Catarina-Brazil, cattle presented high prevalences of *B. bovis* (29%) and *B. bigemina* (16%) [8]. Similarly, water buffaloes showed prevalences of 16.2% for *B. bovis* and 24% for *B. bigemina* in the county of Papaloapan, state of Veracruz, Mexico [9].

The presence of ticks and tick-associated diseases generate economic losses not only due to decreased production, mortality, and control [10], but also in animal trade [2]. *Rhipcephalus microplus (R. microplus)* is described as the main vector of *Babesia* spp. in South America [2,4,11]. For example, in Brazil, in 2011, *R. microplus* caused a decrease in milk and meat production, resulting in economic losses of US$3.24 million [12].

*R. microplus* ticks cohabit with cattle in tropical and subtropical areas, resulting in the presence of persistently infected cattle [3]. In Europe, climatic conditions such as humidity and temperature would limit the distribution of ticks such as *I. ricinus* [13]. In Central and South America, optimal environmental conditions such as climate, soil and cattle biotypes favor *R. microplus* populations as well as the infection rate of cattle and ticks [4].

Studies in Latin America have shown that climatic conditions and altitude influence indirectly the distribution and prevalence of *Babesia* spp. In Bolivia, cattle at altitudes of 300 to 1300 m above sea level have a higher risk of babesiosis than those at altitudes of 700 to 2000 m above the sea level (m.a.s.l) [14]. In Colombia, *Babesia* spp. infection values are higher in cattle, buffaloes, and ticks, during and at the end of the rainy season [7]. Recent findings suggest that “climate change” could be modifying the habitats of ticks, evidencing their presence in areas where they have not been traditionally reported, increasing the possibilities of babesiosis outbreaks [1].

Ecuador is located in the equatorial zone and has several bioclimatic levels ranging from over 2000 m.a.s.l (Andean Cordillera) to lowlands at sea level with tropical climates [15], which facilitate the presence of ticks, becoming a problem in cattle production systems. The presence of ticks was found in 88% of cattle farms in the province of Santo Domingo de los Tsáchilas, with a predominance of *R. microplus* (83%) and *Amblyomma cajennense sensu lato* (s.l.) (21%) [16]. In addition, molecular tests as Polymerase Chain Reaction (PCR) and quantitative polymerase chain reaction (qPCR) demonstrated the presence of *B.* *bovis*, *B. bigemina,* and mixed infections in ticks (*R. microplus*) [16,17,18]. On the contrary, the use of a PCR test using ribosomal 18s as a target (PCR) with primers described by Carret et al., 1999 [19], did not demonstrate the presence of *Babesia* spp. in cattle in the province of Pastaza, in the Amazon region of Ecuador, since this is a tick-free zone [20].

Since there is no national program for the control of ticks and tick-borne diseases in Ecuador, producers use different strategies, products, and doses for the treatment against ticks, which has generated problems of resistance to acaricides [16,21].

The aim of this study was to demonstrate the detection and molecular characterization of *Babesia* spp. in cattle from two study areas (coastal area in the province of Manabi; and mountain area in the province of Pinchincha—study of a clinical outbreak), as possible evidence of the adaptation of the vector and the disease to high altitude climates.

## 2. Results

In the El Carmen area, 18.94% (50/264) of the cattle were positive for *Babesia* spp. by PCR. Of the 264 cattle, 14.77% (39/264) were positive for *B. bovis* and 4.17% (11/264) for *B. bigemina*. Of the 143 cattle from the Quito farm, 20.28% (29/143) were positive to *Babesia* spp. by PCR, and 14.69% (21/143) were positive to *B. bovis* and 5.59% (8/143) to *B. bigemina* using restriction enzyme analysis (Table 1).

In El Carmen, 85.61% (226/264) of the total animals sampled were females. For the distribution of animals by age, information was only available for 183 animals. Of the animals positive for *Babesia* spp., 42.86% were found in the 10 to 18 months age group. Regarding the distribution of positive animals, there was no significant difference for sex (Fisher’s Exact test; *p*-value = 0.38) and age group (Fisher’s Exact test; *p*-value = 0.48). *R. microplus* ticks were present in 21.59% (57/264) of the cattle.

Of the total number of animals (*n* = 143) sampled from Cantón Quito, 95.10% (136/143) were females. As for the age distribution, there was a significant difference (Fisher’s Exact test; *p*-value = 0.004). There were 72.41% (21/70) of the *Babesia* spp. positive animals in the 19 to 36 months age group and no positive animals were found in the 0 to 9 months age group (Table 2). No significant difference was found in relation to sex (Fisher’s exact test; *p*-value = 0.35) since the number of males was low (5%), thus this variable was not an explanatory factor (Table 2).

In Quito, three cattle showed symptoms at the time of the visit to the farm, two of which were positive for *B. bovis*. The three animals were females and had been separated from the herd because they presented symptoms such as decay, prostration, and temperature. They received treatment based on Inmidocarp (Imicar^®^, Imidocarb dipropionate 12 g), using 2.5 mL/100 kg of weight. At the time of the inspection, the presence of *R. microplus* ticks was found in the three animals and clinical and zootechnical information was collected (Table 3).

In the examination of the rest of the animals (140), no cattle had placket cell volume (PCV) values less than or equal to 24%, but 2 animals had a body temperature of 39.3 °C and 39.9 °C and these animals were positive for *B. bovis*.

Both Quito and El Carmen were exclusively dairy farms. In El Carmen, there were animals with crossbreeds of *Bos indicus* (Brahman, Gyr, Nelore) and *Bos taurus* (Brown suis, Hosltein, Charolais). In Quito, the animals were *Bos taurus* and there were crossbred animals of Brown suis, Hosltein and Jersey breeds.

### Phylogenetic Analysis

**Figure 1:**
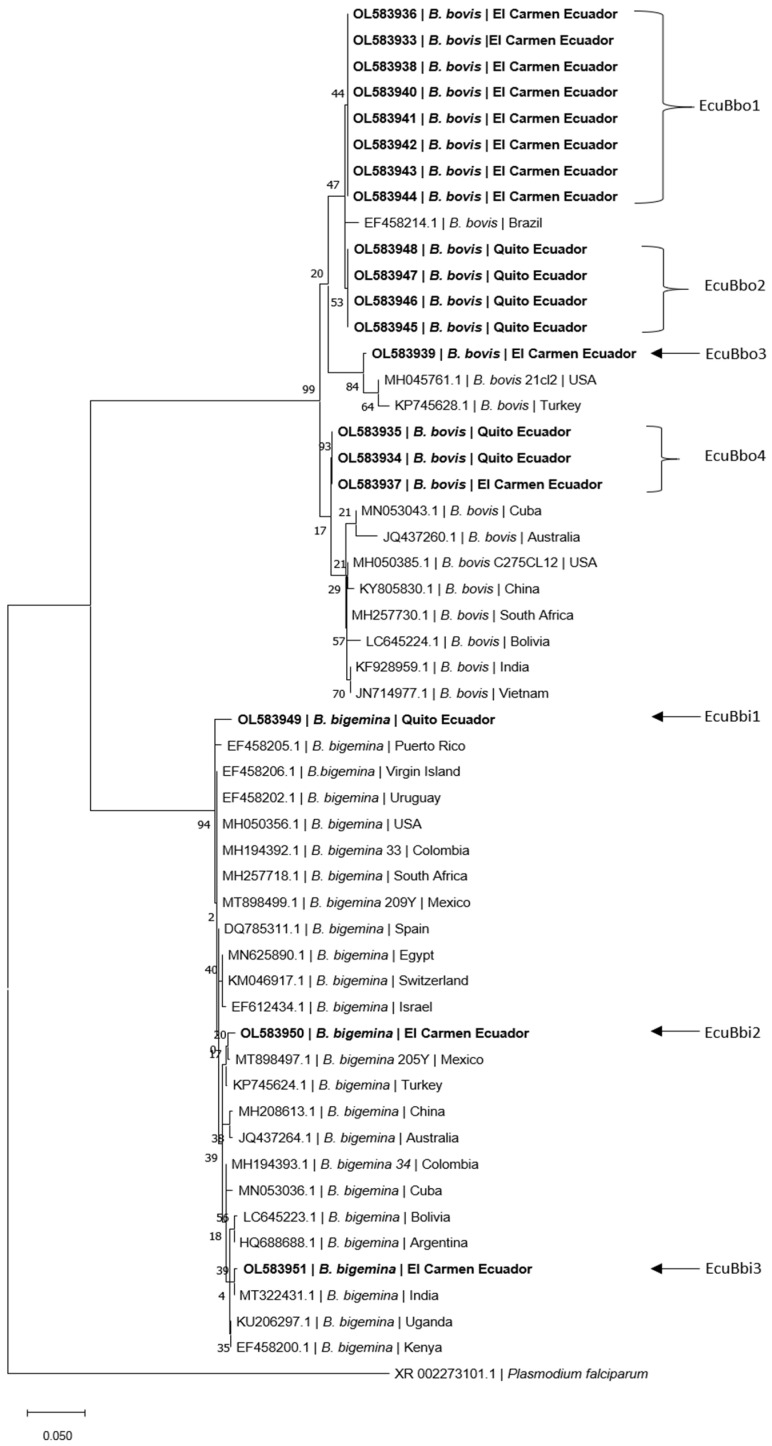
Maximum likelihood, 3-parameter Tamura [22] phylogenetic tree of the 18s rRNA gene sequences of *B. bovis* and *B. bigemina* from Ecuador. It involved 54 nucleotide sequences and a total of 519 positions in the final data set. The following terminology was used to identify the isolates: Ecu = Ecuador, Bbo = *B. bovis*, Bbi = *B. bigemina* and the isolate number. Taxa names consist in GeneBank code/isolates of *Babesia* spp./geographical origin.

Of the consensus sequences (*n* = 19) evaluated in BLAST, 10 samples from El Carmen and 6 from Quito showed similarity to the 18s rRNA of *B. bovis*, while 2 samples from El Carmen and 1 from Quito had similarity to *B. bigemina* (Figure 1).

In El Carmen, of the 10 sequences identified as *B. bovis*, 8 were identical (EcuBbo 1), while the remaining 2 (EcuBbo 2 and EcuBbo 3) differed from each other, as well as from isolate EcuBbo 1. The two sequences of *B. bigemina* were also different from each other (EcuBbi 1 and EcuBbi 2). In Quito, of the six sequences identified as *B. bovis*, four sequences were identical forming the EcuBbo 4 isolate and the remaining two sequences were identical to the EcuBbo 2 isolate from El Carmen. The sequence of *B. bigemina* formed an isolate EcuBbi 3 that differs from El Carmen sequences (Table 4).

## 3. Discussion

In South America, *R. microplus* is widely distributed in the Mesoamerican corridor towards Venezuela and Colombia, and in southern Brazil and Argentina [23]. In Ecuador, data on tick ecology and distribution information are scarce [24]. The present study would be the first finding of *Babesia* spp. and *R. microplus* in a high altitude area, although the farm in Quito is not a preferred area for the tick due to its climatological and altitudinal characteristics [25]. Possibly, in this zone, the environmental temperature influences the adaptation process of the tick where the development phase can be extended (120 days) at temperatures of 18 °C [25]. Due to climate change in recent years, the abundance and distribution of ticks is increasing to new areas [26,27]. In India, ticks on domestic animals are absent at altitudes above 1900 m.a.s.l. However, in closer countries, such as Colombia, *Amblyomma cajennense* is recorded at a maximum altitude of 1771 m.a.s.l. [28]. For *Ixodes* spp., there are records of up to 2410 m.a.s.l. in Venezuela [29].

However, climate change alone should not be a determining factor in tick distribution. Other factors such as land use change [30], as well as the mobility of cattle, can also play an important role in the introduction of ticks to new areas and therefore the introduction of diseases such as *Babesia* spp. [26]. In the city of Quito, cattle arrive from various regions of the country to be butchered in slaughter centers, many of them are carriers of ectoparasites. This mobility without any control can introduce ticks to the pastures occasionally, which would be causing an enzootic instability for hemoparasites [25].

Previous studies in Ecuador demonstrated the presence of *Babesia* spp. in ticks, but not in cattle [16,18]. Our study shows for the first time *B. bovis* and *B. bigemina* in two geographical areas El Carmen and Quito, with a prevalence of 18.93% and 20.28%, respectively for *Babesia* spp. In both regions, *B. bovis* is more prevalent than *B. bigemina*. This is similar to those found in Brazil where a prevalence of 16% for *B. bigemina* and 29% for *B. bovis* was reported in cattle [8], but differs from that found in Colombia where a prevalence of 24.2% for *B. bigemina* and 14.4% for *B. bovis* was recorded [7]. Usually, tick infection rates are lower for *B. bovis* than for *B. bigemina* [2], which may allow tick survival and therefore favor transmission [31]. Moreover, *B. bovis* has a strong ability to survive against host immune pressure [32] and therefore, in the two study areas, *B. bovis* is more predominant. *B. bovis* and *B. bigemina* species can persist for several months and years with a low level of parasitemia in the host, which allows the long-term maintenance of the parasite in the environment and therefore the presence of persistently infected asymptomatic animals [31]. At the level of the bovine population, this condition originates a state called enzootic equilibrium, where no sick animals are observed [2], which is possibly what happened in the area of El Carmen. In the case of Quito, where clinically sick animals were observed, it is likely that there was a recent introduction of the parasite in the area, together with the presence of *R. microplus*, which resulted in the appearance of the outbreak, showing that this area is not in enzootic equilibrium. This imbalance in the host–parasite relationship would lead us to think that the presence of Babesiosis is relatively new in the area.

In South America, most of the outbreaks have been associated with *B. bovis* [4], which is consistent with what was found in the province of Pichincha, where symptomatic animals positive for *B. bovis* were found; this may be due to a delayed, inadequate, and insufficient immune response by the host [31], and to the pathogenicity of the parasite [4].

The age of cattle is correlated with the clinical stages of Babesiosis [2,5,31], so that young animals between 3 and 9 months are more resistant than adult animals [2]; in the Pichincha area the animals with symptomatology were older than 15 months of age. On the other hand, 42.18% of the asymptomatic animals in the El Carmen area belonged to the 10 to 18 months age group, and 28.57% were in the 0 to 9 months age group. Because, in El Carmen, the young animals are the ones that are mostly infected, these animals acquire early immunity, developing what is known as concomitant immunity [5,7] and for this reason, clinical cases are rare. In Quito, 30% belonged to the age group of 19 to 36 months that were positive for *Babesia* spp. These animals were of reproductive age and were inseminated for the first time. In Brazilian buffaloes, pregnant females had the highest infection rate, which was associated with hormonal and immunological changes [33]. The PCV analysis in all PCR positive animals in Quito was normal, excluding one with clinical finding. This is in agreement with what was found in Colombia, where the prevalence of *B. bigemina* and *B. bovis* was higher in animals with normal PCV values [34]. The breeds *Bos taurus* are associated with the presence of Babesiosis [2,31,35]. In the area of Manabí, the animals sampled were *Bos taurus* and *Bos indicus* crossbreeds, and in the area of Pichincha, they were *Bos taurus*, so that crossbreeding with *Bos taurus* in the study areas increases the probability of being infected by the *R. microplus* and therefore, of acquiring Babesiosis [2].

The phylogenetic analysis of *B. bovis* and *B. bigemina* in the two zones of Ecuador was grouped with sequences from other countries of South America, North America, Asia, Africa, and Oceania; this grouping has also been evidenced in isolates from the Brazilian Pantanal [35]. This suggests that most of the sequences found are not exclusive to Ecuador. This analysis also revealed four isolates of *B. bovis*: three isolates (EcuBbo 1, EcuBbo 2 and EcuBbo 3) in the area of El Carmen and two isolates (EcuBbo 2 and EcuBbo 4) in the area of Quito, showing that EcuBbo 2 is present in both areas, indicating that the parasites were transferred from the Ecuadorian coast to the temperate zone of the Sierra (i.e., Quito), possibly due to the mobility of animals. On the other hand, for *B. bigemina* in El Carmen, two isolates were determined (EcuBbi 1 and EcuBbi 2) and in the Quito area, only one isolate EcuBbi 3 was identified, none of which was similar between the two zones. Thirty percent of the genetic diversity of *Babesia* spp. is associated with genetic differences between isolates in different geographic regions [36], but this diversity could also be associated with other factors such as the introduction of strains from different geographic locations, diversity of biological and mechanical vectors [7].

When observing the phylogenetic study carried out from the sequences downloaded from BLAST and comparing them to the 19 samples analyzed in this work, two large clades are observed, in the upper branches are the *B. bovis* and in the lower *B. bigemina*. Of the 19 samples evaluated in this study, we identified 7 different genotypes called EcuBbo1 to EcuBbo4 for those similar to *B. bovis* and EcuBbi1 to EcuBbi3 for those similar to *B. bigemina*.

For *B. bigemina*, the three genotypes are slightly different from each other and, in turn, similar to other genotypes described in Latin America and the world; for example, EcuBbi1 migrates very close to isolates from Puerto Rico, Virgin Island, Uruguay, etc. However, EcuBbi3 is similar to isolates from Cuba, Bolivia, and Argentina, but is closer to an isolate from India (see Figure 1).

On the other hand, when considering the genotypes similar to *B. bovis* identified as EcuBbo in this work, it can be highlighted that the similarity between them is much lower, finding that under the EcuBbo1 genotype, there are eight specimens identical to each other and all of them are from Carmen and equally similar to a Brazilian isolate. Four individuals conformed to the genotype identified as EcuBbo2 and all of them came from Quito (2469 m.a.s.l.). The EcuBbo3 isolate forms a unique genogroup that together with the two previous groups constitutes a single clade where sequences from Brazil, USA, and Turkey are found.

Very curiously, the EcuBbo4 group is constituted by three individuals, one comes from Carmen and the other two from Quito. This demonstrates the genetic closeness of both isolates, making clear the probability that the outbreak occurred in the bovines of Quito had its origin in babesiosis from the coastal region, in Ecuador itself.

## 4. Materials and Methods

### 4.1. Study 1—Area of the Province of Manabí

**Figure 2:**
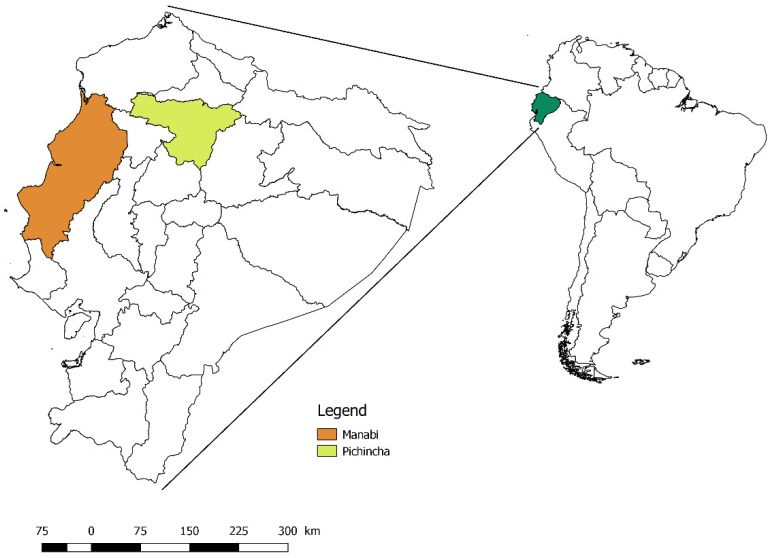
Location of the provinces of Manabí and Pichincha in Ecuador.

Between March–April 2016, this study aimed to study bovine babesiosis in the parish of San Pedro de Suma the canton of El Carmen in the province of Manabí, in the coastal region of Ecuador, because this area concentrates 21.95% of the national bovine production [37] (Figure 2). The area belongs to the ecosystem: seasonal lowland evergreen forest of the Equatorial Chocó; with altitudes from 0 to 300 m.a.s.l., it has an infratropical thermotype and a humid bioclimate [15], with average temperatures of 27.2 °C (min 20.2 °C–max 34.4 °C), and average relative humidity of 79% (min 40%–max 92%) [38].

We collected blood samples from cattle (*n* = 264) on twenty farms (*n* = 20). Zootechnical information such as age and sex were collected from each animal. Age was distributed in four groups: 0 to 9 months, 10 to 18 months, 19 to 36 months, and older than 48 months.

For the sampling design, a database of the farms (*n* = 100) existing in the parish of San Pedro de Suma from El Carmen, was obtained by interviewing the President of the Producers’ Association. Depending on the number of existing cattle, the farms were categorized into: small (less than 20 cattle), medium (21 to 70 cattle), or large (more than 70 cattle). The sampled cattle came from small (*n* = 10), medium (*n* = 6), and large (*n* = 4) farms, which were randomly selected from the database. The blood samples were obtained from a random sampling in a percentage depending on the category of the farm: small (minimum 50% of bovines), medium (minimum 25%), and large (minimum 13%), without the existence of exclusion variables.

### 4.2. Study 2—Area of the Province of Pinchincha

In May 2020, an outbreak of Babesiosis was reported in cattle on a farm in the parish of Conocoto (Canton Quito), 18 km from Quito, in the province of Pichincha in the Sierra Region (Figure 2). The area belongs to the evergreen montane shrubland ecosystem of the northern Andes. It corresponds to the Montane bioclimatic floor, with altitudes of 2000–3000 m.a.s.l, and a mesotropical thermotype; with a bioclimate as humid with an average relative humidity of 74% (min 27%–max 94%) [15] (Ecosystem Classification Systems of Continental Ecuador, 2013) and characterized by temperatures min 14 °C–max 21 °C [39]. The altitude of the farm is 2469 m.a.s.l.

We collected blood samples from cattle (*n* = 143) of this outbreak, and zootechnical information such as age and sex were collected; furthermore, clinical parameters as PCV (packed cell volume) and rectal temperature were recorded in order to identify another possible sick animal.

### 4.3. Collection and Analysis of Blood Samples

In the two study areas, blood samples in tubes without (VANTUBO^®^) and with anticoagulant (EDTA-K2 0.75 mm × 25 mm VACUTECH^®^) were collected from each animal by puncture of the coccygeal vein.

### 4.4. Packed Cell Volume Determination

The packed cell volume value (≤24% is related to anemia) [40] was determined from each blood sample (EDTA tube); for this, a fraction of blood was transferred to a capillary with heparin (TECNAN^®^) and centrifuged (TG12M Madell Techinology Corporation, Riverside, CA, USA) at 10,000 rpm for 5 min and its reading was performed according to the usual procedures [40].

### 4.5. DNA Extraction

DNA extraction from blood was performed following the protocol described by Tana-Hernandez et al., (2017) [41]. It was quantified by UV spectrophotometry in NanoDrop 2000 equipment (Thermo Fisher Scientific, Waltham, MA, USA), obtaining an average concentration of 114.7 ng/mL, and DNA integrity was verified by electrophoresis in a 0.8% agarose gel.

### 4.6. RFLP-PCR for the Detection of the 18s Fragment for Babesia spp.

For the detection of Babesia, a conventional PCR was performed using as specific primers: PIRO A (5’-AATACCCAATCCTGACACACAGGG-3’) and PIRO B (5’-TTAAATACACGAATGCCCCCCCAAC-3’), which partially amplify the 18s rRNA gene of *Babesia* spp. [42]; subsequently, the amplicons were cut with restriction enzymes, following the protocol described by Figueroa (2014) [43], sing HpaII (Thermo Scientific, Waltham, MA, USA) to identify *B. bovis* and BoxI (Thermo Scientific, Waltham, MA, USA) for *B. bigemina*, according to the conditions of the commercial house.

PCR products from positive samples were purified using the Wizard^®^ SV Gel and Clean-Up System (Promega, Wisconsin, Madison, WI, USA), and sent for sequencing to MACROGEN (Seoul, Korea).

### 4.7. Analysis of the Sequence Obtained

In order to obtain the sequences to carry out the present work, one sample for each farm (*n* = 20) (the most prominent amplicon on each farm) was sent three times to sequence, of which we only worked with those sequenced with higher quality indices of more than 90%. In the case of the Pichincha farm (outbreak), 17 samples were sequenced; all of them exceeded the required quality index.

These sequences were assembled using the Clustal W and BioEdit programs to obtain a consensus sequence for each of the samples. The consensus sequences were up-loaded to GenBank (Accession numbers: OL583933, OL583934, OL583935, OL583936, OL583937, OL583938, OL583939, OL583940, OL583941, OL583942, OL583943, OL583944, OL583945, OL583946, OL583947, OL583948, OL583949, OL583950, OL583951). Subsequently, the similarity and homology of the consensus sequences were evaluated with the database of the National Center for Biotechnology Information (NCBI) using the Basic Local Aligment Search Tool (BLAST). Evolutionary analyses were performed in MEGA X [44], using the maximum likelihood method and Tamura’s 3-parameter model [23]. For this analysis, 34 partial 18s rRNA sequences of *B. bovis* and *B. bigemina* from Genbank and one sequence of *Plasmodium falciparum* as an outgroup were used.

### 4.8. Statistical Analysis

Age and sex were the only two variables studied, and the significance of the difference in the distribution of the results was analyzed by the Fisher’s Exact test, due to the low number of observations.

The PCV and the breed of the bovines were not analyzed as a possible explanatory factor, because in study 1 (El Carmen), complete information on these variables was not obtained, whereas in study 2 (Quito), complete information on these variables was obtained and the cattle had crosses with the Holstein, Brown Swiss, and Jersey breeds.

## 5. Conclusions

There is little information on the abundance and distribution of *R. microplus* in Ecuador. The presence of bovine Babesiosis in the area of Quito suggests that this tick can reach increasingly higher altitudes; this may be due to climatic factors, but also to the mobility of animals carrying ticks without any restriction or control.

This study evidenced for the first time the presence of *Babesia* spp. in the area of El Carmen located in the coastal region and in the area of Quito located in the highlands region. In addition, we found two circulating species *B. bovis* and *B. bigemina* in cattle. *Babesisa bovis* was the most predominant in both study zones; this species is associated with the outbreak reported in the Quito farm where symptomatic animals were found.

The sex of the cattle in both the El Carmen and Quito areas was not an explanatory factor. Age was an explanatory factor associated with the presence of Babesiosis in cattle in Quito, in which symptomatology was evidenced. It is possible that Babesiosis is entering the area, while in El Carmen, it is likely that this parasite is in enzootic equilibrium.

This would be the first molecular characterization of *B. bovis* and *B. bigemina* in cattle in two geographic zones of Ecuador, finding similarities in both zones, which would indicate that the hemotropics of the Quito zone come from the coastal region, possibly introduced due to animal mobility.

## Figures and Tables

**Table 1:** Presence of *Babesia* spp., in the canton El Carmen province of Manabí and in the canton Quito province of Pichincha, Ecuador.

Farm Code	Total Animals Present on the Farm	Sample		Positive *Babesia* spp.		Positive *B. bovis*		Positive *B. bigemina*	
				PCR 18s		Restriction Enzymes		Restriction Enzymes	
		No	% by Farm	No	%	No	%	No	%
**El Carmen-Manabí Zone**									
F-1	8	8	100	1	12.5	1	12.5	0	0
F-2	21	5	23.8	0	0	0	0	0	0
F-3	16	7	43.8	0	0	0	0	0	0
F-4	194	39	20.1	0	0	0	0	0	0
F-5	43	6	14	0	0	0	0	0	0
F-6	152	17	11.2	0	0	0	0	0	0
F-7	6	4	66.7	2	50	2	50	0	0
F-8	60	20	33.3	7	35	4	20	3	15
F-9	112	36	32.1	8	22.2	6	16.7	2	5.6
F-10	38	8	21.1	0	0	0	0	0	0
F-11	16	16	100	7	43.8	6	37.5	1	6.3
F-12	165	28	17	2	7.1	2	7.1	0	0
F-13	17	9	52.9	0	0	0	0	0	0
F-14	27	25	92.6	6	24	3	12	3	12
F-15	8	4	50	0	0	0	0	0	0
F-16	5	5	100	1	20	0	0	1	20
F-17	2	2	100	0	0	0	0	0	0
F-18	17	6	35.3	3	50	3	50	0	0
F-19	27	12	44.4	9	75	8	66.7	1	8.3
F-20	9	7	77.8	4	57.1	4	57.1	0	0
All farms	943	264	28	50	18.93	39	14.77	11	4.17
**Quito-Pichincha Zone**									
F-1	164	143	87, 20	29	20, 28	21	14, 69	8	5, 59

Legend: No: number; %: percentage; PCR: Polymerase chain reaction. In bold are the totals in column identified with “No” and the average in column with %, respectively.

**Table 2:** Distribution of samples and analysis of risk factors: sex and age groups for bovine *Babesia* spp. in Ecuador.

Variables	Sample		Positive		Fisher’s Exact Test (*p*-Value)
	Number	%	Number	%	
**El Carmen-Manabí Zone (*n* = 264)**					
Sex					0.38
Male	38	14.39	5	13.16	
Female	226	85.61	45	19.91	
Age group					0.48
0 to 9	21	7.95	6	28.57	
10 to 18	7	2.65	3	42.86	
19 to 36	18	6.82	4	22.22	
>36 months	137	5189	29	21.17	
ND	81	30.68	8	9.88	
**Quito-Pichincha Zone (*n* = 143)**					
Sex					0.35
Male	7	4.9	0	0	
Female	136	95.1	29	21.32	
Age group					0.004
0 to 9	26	18.18	0	0	
10 to 18	27	18.88	4	13.79	
19 to 36	70	48.95	21	72.41	
>36 months	20	13.99	4	13.79	

Legend: The % value corresponds to the total number of animals sampled in the area or in each farm; ND, not dertermined.

**Table 3:** Clinical information of three symptomatic bovines in the farm of Cantón Quito.

No	Farm Code	Age	Weight	T	PCV	Heart Rate	Respiratory Rate	Other Clinical Findings	Positive *B. bovis* by PCR
1	425	12	232	39.5	19	84	47	Pale mucous membranes, swollen cervical glands	−
2	433	15	199	39	16	85	64	Salivation, pale mucous membranes, jaundice	+
3	38	15	202	40	29	100	57	Swollen groin glands	+

Legend: Age in months; Weight in kilograms; T°: temperature in Celsius degree; PCV: packed cell volume (values less than or equal to 24% is related to anemia); PCR: polymerase chain reaction; −: negative; +: positive.

**Table 4:** Percentage of identity of *B. bovis* and *B. bigemina* species found in El Carmen and Quito.

Study Areas	Ecuadorian Code	Isolated	Species	Total Score	Identity Percentage	Query Coverage	No. Access Gen Bank
El Carmen (province of Manabi)	M5	EcuBbo 1	*B. bovis*	684	100%	98%	OL583933
	M92						OL583936
	M100						OL583938
	M149						OL583940
	M154						OL583941
	M187						OL583942
	M245						OL583943
	M279						OL583944
	M97	EcuBbo 2	*B. bovis*	684	100%	98%	OL583937
	M103	EcuBbo 3	*B. bovis*	678	99.73%	98%	OL583939
	M232	EcuBbi 1	*B. bigemina*	717	99.74%	99%	OL583950
	M260	EcuBbi 2	*B. bigemina*	721	99.75%	100%	OL583949
Quito (province of Pinchincha)	85	EcuBbo 2	*B. bovis*	684	100%	98%	OL583934
	86						OL583935
	310	EcuBbo 4	*B. bovis*	678	99.73%	98%	OL583946
	300						OL583945
	314						OL583947
	315						OL583948
	260	EcuBbi 3	*B. bigemina*	701	98.98%	99%	OL583951

Legend: The total score is the sum of alignment scores of all segments from the same database sequence that match the query sequence (calculated over all segments), the percent identity is a number that describes how similar the query sequence is to the target sequence, and the query cover is a number that describes how much of the query sequence is covered by the target sequence (taken from the National Center for Biotechnology Information, NCBI).

## Data Availability

The data that support the findings of this study are available from the corresponding author upon reasonable request.
